# Coronavirus Disease 2019 (COVID-19) Associated With Febrile Status Epilepticus in a Child

**DOI:** 10.7759/cureus.9840

**Published:** 2020-08-18

**Authors:** Madhuradhar Chegondi, Harsh Kothari, Swathi Chacham, Aditya Badheka

**Affiliations:** 1 Pediatrics, University of Iowa Stead Family Children's Hospital, Iowa City, USA; 2 Division of Pediatric Critical Care Medicine, Stead Family Children's Hospital, Iowa City, USA; 3 Pediatrics, All India Institute of Medical Sciences, Rishikesh, IND; 4 Pediatrics, University of Iowa Hospitals and Clinics, Iowa City, USA; 5 Pediatrics, Stead Family Children's Hospital, Iowa City, USA

**Keywords:** covid-19, sars-cov-2, febrile status epilepticus, child

## Abstract

Infection associated with the novel severe acute respiratory syndrome coronavirus 2 (SARS-CoV-2) has been named coronavirus disease 2019 (COVID-19). The emerging literature suggests that SARS-CoV-2 infection affects children of all age groups. COVID-19 as a cause of febrile seizures and status epilepticus is not yet reported in children. We report the case of a two-year-old child who presented to our pediatric intensive care unit with febrile status epilepticus and was diagnosed to have COVID-19 infection. The child recovered fully and was discharged home after three days.

## Introduction

The novel severe acute respiratory syndrome coronavirus 2 (SARS-CoV-2) emerged in Wuhan City, Hubei Province, China, in December 2019. Infection associated with it was named Coronavirus disease 2019 (COVID-19). Ever since the first case was identified, COVID-19 rampantly spread all over the world and resulted in more than 15 million cases and 619,000 deaths globally [1]. The emerging literature suggests that the SARS-CoV-2 infection can affect children, including all age groups, predominantly males, and cause milder disease compared to adult patients [2,3]. In the United States, 1.7% of all confirmed COVID-19 cases are children (≤18 years), and 5%-20% of them were hospitalized [4]. Recently, two pediatric COVID-19 cases were reported with afebrile seizures [5,6]. However, COVID-19 as a cause of febrile seizures and status epilepticus has not yet been reported. We report the case of a two-year-old child who presented to our pediatric intensive care unit (PICU) with febrile status epilepticus and was diagnosed to have COVID-19 infection.

## Case presentation

A two-year-old, previously healthy female was transferred from a community hospital emergency department (ED) to our PICU with new-onset febrile seizure that presented as status epilepticus. The child started with a tactile fever and reduced oral intake a few hours earlier on the day of admission. Her parents took her to the local ED, where she was noted to have a temperature of 39.9°C with generalized tonic-clonic seizures. An intravenous (IV) access was established, and the patient received a dose of lorazepam. Furthermore, she was given four more doses of lorazepam and levetiracetam to abort seizures. After that she was noted to have respiratory depression and oxygen desaturation. She was endotracheally intubated with propofol and rocuronium and placed on a mechanical ventilator. Given the ongoing COVID-19 pandemic, appropriate personal protective measures were taken during the endotracheal intubation. The patient also received a dose of IV acetaminophen and one normal saline bolus 20 mL/kg for borderline blood pressure post-intubation. Infectious workup was pursued in the setting of febrile seizure, and screening labs were obtained. Blood culture and urine culture were collected, and a dose of ceftriaxone 50 mg/kg was given. Ethanol, salicylate, and acetaminophen levels were normal. Her urine drug screen was negative. Her complete blood count showed leukocytosis with a white cell count of 17.69 × 10^9^/µL, neutrophil predominance, and microcytic anemia. She had no thrombocytopenia or lymphopenia. Her C-reactive protein was mildly elevated at 1.4 mg/dL; her electrolytes, blood glucose, urea, creatinine, and liver function test were unremarkable. Her lactic acid was elevated at 5 mmol/L. The urine analysis showed mild ketones and protein without evidence of urinary tract infection. The respiratory viral panel was negative. COVID-19 testing was sent, and the result was pending. A CT image of the brain was normal. Per the parent report, the child is fully immunized and did not have nasal congestion, cough, or gastrointestinal symptoms. The parents denied seizures or COVID-19 exposure in the family. However, her father had cough and congestion for the previous few days and was not tested for COVID-19. 

**Figure 1:**
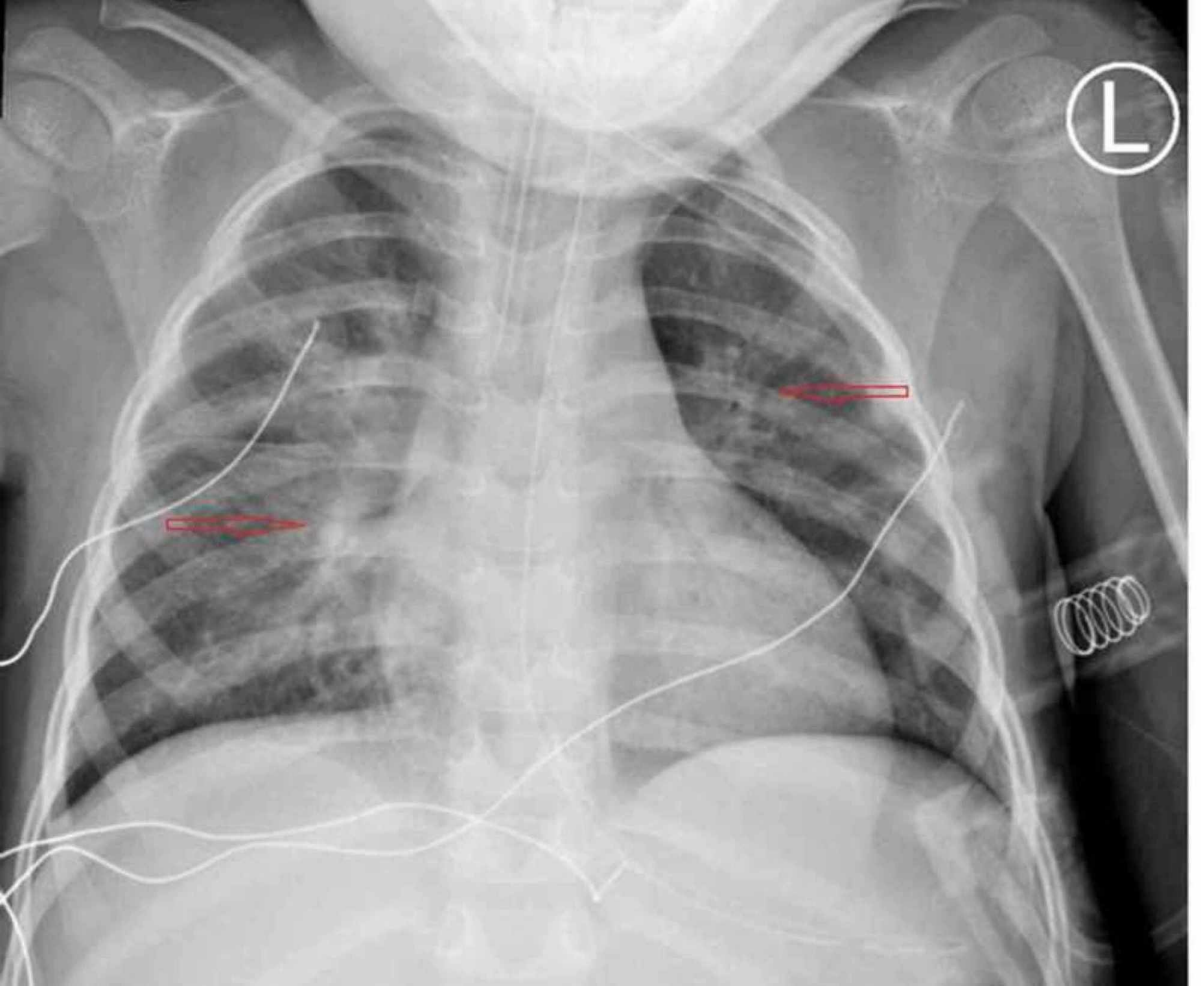
Chest X-ray showing bilateral patchy atelactasis

The patient was transferred to our PICU on propofol infusion. Upon arrival to PICU, she was appropriately sedated on the mechanical ventilator, and was normothermic, with stable hemodynamics. The child was able to move all extremities in deep stimulation, and her bilateral pupils were equal and reactive to light. Admission blood gas and repeat lactate were normal, and her chest X-ray showed patchy infiltrates bilaterally (Figure 1).

A repeat nasopharyngeal swab using real-time polymerase chain reaction for COVID-19 was sent and returned positive. Within two hours following admission, the child had a recurrence of seizure, which was aborted with a dose of lorazepam and a loading dose of fosphenytoin. The neurology service was consulted, and the patient was started on maintenance levetiracetam. The child was kept on continuous electroencephalogram (cEEG) monitoring for 24 hours. cEEG showed generalized slowing without recurrence of seizures. A lumbar puncture was performed, and cerebrospinal fluid (CSF) was sent for analysis. CSF showed one nucleated cell per mm^3^, less than 500 red blood cells per mm^3^, protein 12 mg/dL, and glucose 86 mg/dL. The CSF viral meningitis and encephalitis panel was negative. Due to testing restrictions, we were unable to send for COVID-19 testing in CSF or serology. The patient was continued on ceftriaxone, and vancomycin was added. On day 2 of the PICU stay, she was extubated under deep sedation to nasal cannula oxygen, and then was gradually weaned off to ambient air. She remained afebrile throughout her PICU stay, and antibiotics were discontinued as 48-hour cultures were negative. The child was discharged the following day on levetiracetam maintenance and advised to follow up with the neurology outpatient clinic in two weeks.

## Discussion

The SARS-CoV-2 virus is one of the beta coronaviruses, which also includes severe acute respiratory syndrome virus (SARS-CoV), the Middle Eastern respiratory syndrome (MERS) virus, HCoV-HKU1, and HCoV-OC43 [7]. Alpha coronaviruses include HCoV-229E and HCoV-NL63 [7]. Among human coronaviruses (HCoVs), HCoV-229E, -OC43, -NL63, and -HKU1 strains are endemic worldwide [8]. HCoVs mostly cause upper respiratory tract illness (URTI) and can cause severe lower respiratory tract illness in children with underlying comorbidities. However, in COVID-19 cases, URTI is less frequent [9]. Among pediatric COVID-19 cases in the United States, fever and cough present in 56% and 54% of the cases, respectively, and intensive care admission was required in 1.8% of them [2,4].

In children, the extrapulmonary manifestations of HCoV include acute gastroenteritis, myocarditis, and multi-organ failure [8]. HCoV-associated neurological features include meningitis, encephalitis, acute flaccid paralysis, acute disseminated encephalomyelitis, and refractory status epilepticus [8,10]. Among the coronaviruses, HCoV-HKU1 has been reported as a common cause of febrile seizures in children [11]. Neurological involvement in COVID-19 patients has been described. A retrospective study from China reported that common neurological symptoms in adult patients with COVID-19 include headache, dizziness, and rarely seizures [12]. Karimi et al. reported frequent seizures in a young adult with COVID-19 [13]. In pediatric COVID-19 cases, headache is less frequently reported compared to adult patients [4]. However, as we were writing this case report, we could not find febrile seizures in children that were associated with COVID-19, though afebrile seizures were reported [5,6]. The possible mechanisms of coronavirus-associated neurological manifestations are direct virus invasion and effect of inflammatory mediators [10]. A study by Huang et al. reported that COVID-19 is associated with cytokine storm, and these cytokines might result in neuronal excitability and seizures [9]. It has been shown that HCoV-O43,-229E, and SARS-CoV are neuroinvasive [8,14]. Fatal encephalitis has been described with HCoV-OC43 in immunocompromised children [15,16]. In these children, the CSF analysis was negative, but the brain biopsy RNA sequencing was positive for HCoV-OC43. However, in our index case, CSF was normal, and rapid improvement in sensorium suggests that COVID-19-associated encephalitis is unlikely. Due to limited COVID-19 testing capability, we could not check the CSF for SARS-CoV-2.

## Conclusions

In children, COVID-19 incidence is lower, and severe disease is rare compared to adults. The majority of children with COVID-19 present with cough and fever. Febrile seizures have been described with HCoV infections in children, especially with HCoV-HKU1. Our index case illustrates that SARS-CoV-2 associated COVID-19 can present with febrile seizure and febrile status epilepticus in children.
